# Cost-Effectiveness of Apixaban versus Warfarin in Chinese Patients with Non-Valvular Atrial Fibrillation: A Real-Life and Modelling Analyses

**DOI:** 10.1371/journal.pone.0157129

**Published:** 2016-06-30

**Authors:** Xue Li, Vicki C. Tse, Wallis C. Y. Lau, Bernard M. Y. Cheung, Gregory Y. H. Lip, Ian C. K. Wong, Esther W. Chan

**Affiliations:** 1 Centre for Safe Medication Practice and Research, Department of Pharmacology and Pharmacy, The University of Hong Kong, Hong Kong SAR, China; 2 Department of Medicine, Li Ka Shing Faculty of Medicine, The University of Hong Kong, Hong Kong SAR, China; 3 University of Birmingham Centre for Cardiovascular Sciences, City Hospital, Birmingham, United Kingdom; 4 Research Department of Practice and Policy, School of Pharmacy, University College London, London, United Kingdom; University of Miami School of Medicine, UNITED STATES

## Abstract

**Objectives:**

Many of the cost-effectiveness analyses of apixaban against warfarin focused on Western populations but Asian evidence remains less clear. The present study aims to evaluate the cost-effectiveness of apixaban against warfarin in Chinese patients with non-valvular atrial fibrillation (NVAF) from a public institutional perspective in Hong Kong.

**Methods:**

We used a Markov model incorporating 12 health state transitions, and simulated the disease progression of NVAF in 1,000 hypothetical patients treated with apixaban/warfarin. Risks of clinical events were based on the ARISTOTLE trial and were adjusted with local International Normalized Ratio control, defined as the time in therapeutic range. Real-life input for the model, including patients’ demographics and clinical profiles, post-event treatment patterns, and healthcare costs, were determined by a retrospective cohort of 40,569 incident patients retrieved from a Hong Kong-wide electronic medical database. Main outcome measurements included numbers of thromboembolic and bleeding events, life years, quality-adjusted life years (QALYs) and direct healthcare cost. When comparing apixaban and warfarin, treatment with incremental cost-effectiveness ratio (ICER) less than one local GDP per capita (USD 33,534 in 2014) was defined to be cost-effective.

**Results:**

In the lifetime simulation, fewer numbers of events were estimated for the apixaban group, resulting in reduced event-related direct medical costs. The estimated ICER of apixaban was USD 7,057 per QALY at base-case analysis and ranged from USD 1,061 to 14,867 per QALY under the 116 tested scenarios in deterministic sensitivity analysis. While in probabilistic sensitivity analysis, the probability of apixaban being the cost-effective alternative to warfarin was 96% and 98% at a willingness to pay threshold of USD 33,534 and 100,602 per QALY, respectively.

**Conclusions:**

Apixaban is likely to be a cost-effective alternative to warfarin for stroke prophylaxis in Chinese patients with NVAF in Hong Kong.

## Introduction

Non-valvular atrial fibrillation (NVAF), the most common sustained cardiac arrhythmia, affects 1–2% of the general population worldwide and its prevalence increases with age [1, 2]. As one of the risk factors for stroke and systemic embolism, NVAF is associated with long-term morbidity, impaired quality of life, mortality and considerable financial burden. The healthcare cost for patients with NVAF is estimated to increase 1.6–3.1 fold after the occurrence of thromboembolic events, particularly in the first three years [3]. Therefore, stroke prevention in patients with NVAF is important from both individual and societal perspectives.

Long-term anticoagulation management is required for stroke prophylaxis in patients with NVAF. Traditionally, warfarin (a vitamin K antagonist) has been the most commonly used anticoagulant [4]. However warfarin poses challenges with respect to clinical management due to its narrow therapeutic range and drug metabolism affected by multiple factors such as genetic variables, diet and drug interactions[5]. Apixaban (a novel direct factor Xa inhibitor) was therefore developed as an alternate anticoagulant. A multinational trial of ARISTOTLE (Apixaban for Reduction in Stroke and Other Thromboembolic Events in Atrial Fibrillation) demonstrated apixaban to be superior to warfarin in prevention of ischaemic stroke (21% reduction), bleeding (31% reduction) and mortality (11% reduction) without the need for monitoring [6]. Recent subgroup analysis of the landmark trial showed consistent protective effect of apixaban on stroke, bleeding and mortality in East Asian and non-East Asian patients [7].

In addition to improved clinical efficacy and safety compared against standard treatment, cost-effectiveness is also an important consideration for new treatments in the setting of increased healthcare costs globally. Apixaban has been found to be cost-effective against warfarin in Europe [8–10], Australia [11] and USA [12]. Notably, the current CE literature all focused on Western populations. Although Asians and Westerns share similar risk factor profiles, they might not be the same due to more history of stroke, increased tendency to bleed and suboptimal international normalised ratio (INR) control when treated with warfarin [13]. These factors may have an impact on the cost-effective manner of anticoagulation therapies. Hence, it is important to incorporate local data to evaluate CE in the Asian population. The objective of the present study is to evaluate the cost-effectiveness of apixaban versus warfarin for stroke prophylaxis in Chinese patients with NVAF, from the public institutional perspective in Hong Kong.

## Methods

The research protocol was approved by the Institutional Review Board of the University of Hong Kong/Hospital Authority Hong Kong West Cluster, Hong Kong. No written informed consent was obtained from patients for their clinical records to be used in this study, due to this is a retrospective study based on electronic medical database. All of the patient records/information was anonymized and de-identified prior to analysis.

### Overview

**Fig 1:**
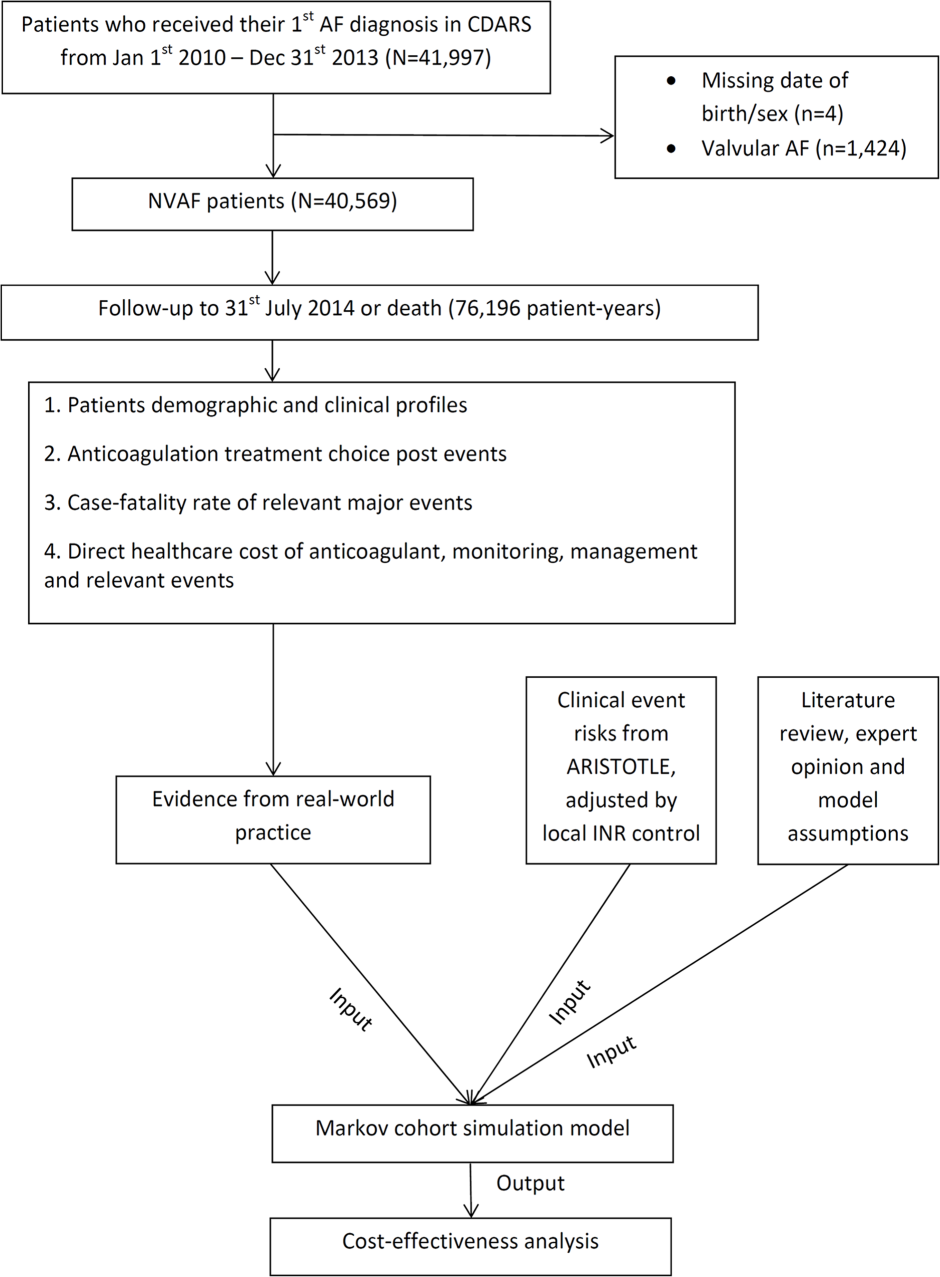
Data sources for Markov cohort simulation. ARISTOTLE: Apixaban for Reduction in Stroke and Other Thromboembolic Events in Atrial Fibrillation; CDARS: Clinical Data Analysis and Reporting System of Hong Kong; NVAF: non-valvular atrial fibrillation.

This study compares the cost-effectiveness of apixaban against warfarin in Chinese patients with NVAF using a Markov model. Data sources and the retrieval procedure are illustrated in **Fig 1.** Lifetime cost and health outcomes were estimated from Markov cohort simulations. A retrospective cohort study was conducted to obtain real-life data for the model inputs. Clinical event risks were based on the ARISTOTLE trial [6] and incorporated by local INR control. Where local data were not available, data retrieved from comprehensive literature review and expert opinion were used. Baseline inputs are listed in **Tables A and B in S1 File**. Model inputs were entered, cross-checked and analysed independently by two researchers.

### Model

A previously developed Microsoft Excel Markov model incorporating 12 health states was adapted to simulate lifetime disease progression in 1,000 patients treated by apixaban (5mg BID) or warfarin (any strength)[14]. The health states modeled include NVAF, ischaemic stroke, systemic embolism, myocardial infarction, bleeding and death. A simplified schematic representation of the model structure and the health states transitions starting from NVAF are shown in **Figures A and B in S1 File**. A detailed description of the model can be found in previous publications [8, 10, 15]. For each treatment group, the number of events in each health state, life years (LYs), quality-adjusted life years (QALYs) and direct healthcare cost were calculated at the end of each model cycle and accumulated across lifetime.

### Population

The target population of the model are patients with NVAF who are suitable for warfarin therapy. Patient profiles were determined by a retrospective cohort analysis of incident patients with NVAF in the Clinical Data Analysis and Reporting System (CDARS). In brief, CDARS is an electronic medical database in Hong Kong that covers a population of over 7 million utilising public healthcare services and accounts for 80% of inpatient records in the territory [16]. De-identified electronic patients’ records, including demographics, diagnoses, procedures, drug descriptions, laboratory tests and date of consultation, hospital admission and discharge, were all imported to CDARS for audit and research purposes.

At the time of the study, CDARS was unable to identify patients with NVAF directly. Target patients were identified by first retrieving patients with documented diagnosis of atrial fibrillation from Jan 1st 2010 to Dec 31st 2013 using ICD-9 diagnosis codes (**Table C in S1 File** for ICD-9 codes used). Among these patients, those with diagnoses of valvular heart disease, hyperthyroidism or had undergone valvular replacement surgery within 1 year prior to the index date were further excluded according to corresponding ICD-9 codes. The follow-up of each patient was commenced from the index date until the occurrence of any clinical events, death or July 31^st^ 2014, whichever came first. This resulted in the identification of 40,569 eligible patients with 76,196 patient-years of follow-up. The key demographic and clinical profiles of these patients are shown in **Table 1.**

**Table 1:** Demographic and clinical profiles of patients with NVAF in CDARS 2010–13.

Characteristics	
***Sample size***	**40,569**
*Demographics*	
Age [median (interquartile range)]	78.9 (69.7–85.6)
Female sex (%)	50.4
***Medical history***	
Prior myocardial infarction (%)	9.2
Prior clinically relevant bleeding (%)	19.3
Prior stroke, transient ischaemic attack, or systemic embolism (%)	20.6
Heart failure or reduced left ventricular ejection fraction (%)	25.3
Diabetes (%)	22.2
Hypertension requiring treatment (%)	76.0
***CHADS***_***2***_ score*	
Distribution (%)	
0–1	41.6
2	24.0
≥3	34.4
Mean (SD)	2.0 (1.5)
***TTR distribution (%)*** ^***†***^	
TTR < 52.38%	61.3
52.38% ≤ TTR < 66.02%	18.1
66.02% ≤ TTR < 76.51%	9.0
TTR ≥ 76.51%	11.6
***Anticoagulation management*, *monitoring and routine care***	
Frequency of INR monitoring (number/month)	1.0
Patients experiencing dyspepsia whilst on warfarin (%)	0.4
Patients requiring annual renal monitoring while on warfarin (%)	29.7

* CHADS_2_: congestive heart failure, hypertension, age≥75 years, diabetes mellitus and stroke

† TTR: time in therapeutic range estimated using the Rosendaal method.

### Risk of clinical events

The clinical event rates of apixaban and warfarin were obtained from the ARISTOTLE trial [6]. As the risk of ischaemic stroke and bleeding associated with warfarin are highly dependent on the quality of INR control [17, 18], the model was developed to adjust the event risks by local INR control. Time in therapeutic range (TTR) distribution was defined as the indicator of INR control and collected from the CDARS retrospective cohort study.

### Fatality and mortality

For thromboembolic and bleeding events, annual case-fatality rates were estimated from the CDARS retrospective cohort study. All-cause mortality was modelled based on life tables in the general population, adjusting by age, gender and the occurrence of NVAF and relevant co-morbidities.

### Anticoagulation management

Healthcare resource consumption regarding anticoagulation management was reflected by the frequency of routine care and monitoring visits, proportion of patients experiencing dyspepsia and proportion of patients requiring annual renal monitoring whilst on treatment from the CDARS retrospective cohort (**Table A in S1 File**).

### Treatment pattern post-events

Treatment patterns post-event were determined from the CDARS retrospective cohort study, published literature or expert opinion wherever appropriate (**Table A in S1 File**). Following all bleeding events, a certain number of patients were assumed to switch to aspirin as second line treatment. The rate of treatment switch (± 90 days of the event index date) was determined by retrieving prescription records from CDARS and literature. The remaining patients were modelled to have their original treatment interrupted for 6 weeks then restarted [19] (i.e. as shown in **Table A in S1 File**, post intracranial haemorrhage, 44% of patients switched to aspirin and the remaining 56% discontinued their original treatment temporarily and restarted the same treatment 6 weeks later). [20]Following occurrence of ischaemic stroke and systemic embolism, all patients were assumed to continue the original treatment as advised by experts.

### Utility

Utility for NVAF was adopted from Ho et al [21], which was the only study to assess the health utility of Hong Kong patients with NVAF on oral anticoagulants. Due to the absence of population-specific utility estimations, a UK-based utility catalogue was adopted for other health states [22]. Marginal disutility was assumed to be associated with INR monitoring when treated with warfarin due to the required blood test [23]. All utility inputs were discounted at 3.5% per year and detailed in **Table B in S1 File**.

### Cost

The analysis was conducted from a public institutional perspective in Hong Kong so only direct medical costs were considered. Costs are presented to the value of US dollars in 2014 (1 USD to 7.75 HKD) and discounted at 3.5% per year according to cost-effectiveness analyses (CEA) guideline [24].

The total cost components are outlined in **Table B in S1 File**. Daily drug costs for warfarin and apixaban were based on local retail prices (internal communication with Bristol-Myers Squibb, Hong Kong, November 2014). Annual monitoring and management costs were determined by multiplying the cost for blood and renal tests per visit by number of clinical visits per patient from the CDARS retrospective cohort. Similarly, acute care costs associated with clinical events were calculated by multiplying daily inpatient charges in public hospital [25, 26] by the average hospital length of stay estimated from the same cohort. Long-term care costs include medication costs only.

### Analyses

We compared the cost-effectiveness of apixaban versus warfarin by assessing the differences in lifetime cost and clinical benefits. Base-case analysis was performed based on model inputs described earlier. Treatment with incremental cost-effective ratio (ICER) less than the willingness-to-pay (WTP) threshold of one local GDP per capita (USD 33,534 in 2014 [27]) for each QALY gained was considered a cost-effective alternative as recommended by the WHO-CHOICE guideline [28].

The effect of uncertainties in the base-case analysis was evaluated by deterministic and probabilistic sensitivity analyses (SA). In deterministic SA, each of the 116 input parameters varied from a predefined range (95% CI or SD, as listed in **Tables A and B in S1 File**) while all the others were constant to project its effect on ICER. All tested parameters were ranked according to ICER variation and the top 15 most influential parameters were presented in Tornado diagrams.

In probabilistic SA, probability distributions were assigned to each parameter (gamma distribution to event rates, beta distribution to utilities and log-normal distribution to cost) and varied simultaneously in 2,000 iterations for the pair-wise comparisons. The cost and health outcomes were further assessed using cost-effectiveness acceptability curves (CEACs). Two willingness-to-pay (WTP) thresholds, one-time and three-times of local GDP per capita (USD 33,534 and USD 100,602 in 2014 [27]) were applied to explore the proportion of acceptable trials for each treatment.

## Results

### Base-case analysis

From a cohort of 1,000 patients over a lifetime, the use of apixaban was predicted to result in 116 fewer NVAF-related events (12 fewer ischaemic strokes, 15 fewer haemorrhagic strokes, 12 fewer other intracranial haemorrhages, 25 fewer other major bleeds, 2 fewer myocardial infarctions) and 31 fewer event-related deaths than patients treated with warfarin in the base-case analysis (**Table 2**). Compared with warfarin, 36 patients need to be treated with apixaban to avoid one additional stroke, and apixaban is the dominant treatment in reducing bleeding events.

**Table 2:** Health and cost outcome estimations in base-case analysis.

	Apixaban	Warfarin	Difference (apixaban–warfarin)
**Number of events (per cohort of 1000)**			
Ischaemic stroke	179	191	-12
Haemorrhagic stroke	21	36	-15
Systemic embolism	17	18	-1
Other intracranial haemorrhage	9	21	-12
Other major bleeds	106	131	-25
Clinically relevant non-major bleeds	190	239	-49
Myocardial infarction	62	64	-2
*Total number of events*	*584*	*700*	*-116*
**Death (per cohort of 1000)**			
Event related death	268	299	-31
Other deaths	732	701	31
**Health outcome (per patient)**			
Life years	6.84	6.67	0.17
Quality adjusted life years	5.06	4.88	0.18
**Cost components (USD per patient)**			
Anticoagulant	3,239	379	2860
Monitoring and management	64	885	-821
Clinical events			
Ischaemic stroke	2,872	3,121	-249
Haemorrhagic stroke	317	560	-243
Systemic embolism	319	330	-11
Other intracranial haemorrhage	31	75	-44
Other major bleeds	503	661	-158
Clinically relevant non-major bleeds	677	869	-192
Myocardial infarction	376	393	-17
Other cardiovascular hospitalisation	3,928	3,825	103
Total event cost	9,023	9,834	-811
*Total lifetime cost*	*12*,*326*	*11*,*098*	*1*,*228*

The cost of treating one patient with apixaban was USD 1,228 more than warfarin (**Table 2**). This was predominantly attributed to the higher drug price of apixaban, in contrast to lower costs related to management, monitoring and clinical events. Compared with warfarin, the net cost, net health benefits and incremental cost-effective ratios of apixaban were detailed in **Table 3.**

**Table 3:** Base-case cost-effectiveness analysis.

Evaluated items	Apixaban vs. Warfarin
Net cost (USD per patient)	1,228
Net QALYs (per patient)	0.18
Net life-years (per patient)	0.17
Incremental cost per QALY gained (USD/QALY)	7,057
Incremental cost per life year gained (USD/LY)	7,181
Incremental cost per stroke avoided (USD/stroke avoided)*	45,481
Incremental cost per bleed avoided (USD/bleed avoided) ^***†***^	23,615

* Stroke included first-ever and recurrent ischaemic and haemorrhagic strokes

***†*** Bleed included first-ever and recurrent haemorrhagic stroke, other intracranial haemorrhage and other major bleeds.

### Deterministic sensitivity analyses

**Fig 2:**
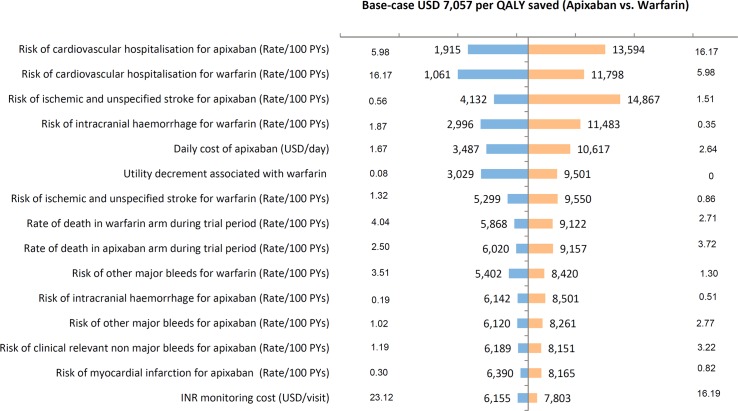
Tornado diagram of apixaban versus warfarin.

As illustrated in **Fig 2**, tested model inputs had limited impact on the ICER. The ICER ranged from USD 1,061 to 14,867 per QALY gained under 116 tested scenarios, all of which were within the threshold of USD 33,534 per QALY (one local GDP per capita). Uncertainties in drug costs, the risk of cardiovascular hospitalisation for both treatments, risk of ischaemic stroke for apixaban and risk of intracranial haemorrhage for warfarin had greatest impact on the ICER.

### Probabilistic sensitivity analyses

**Fig 3:**
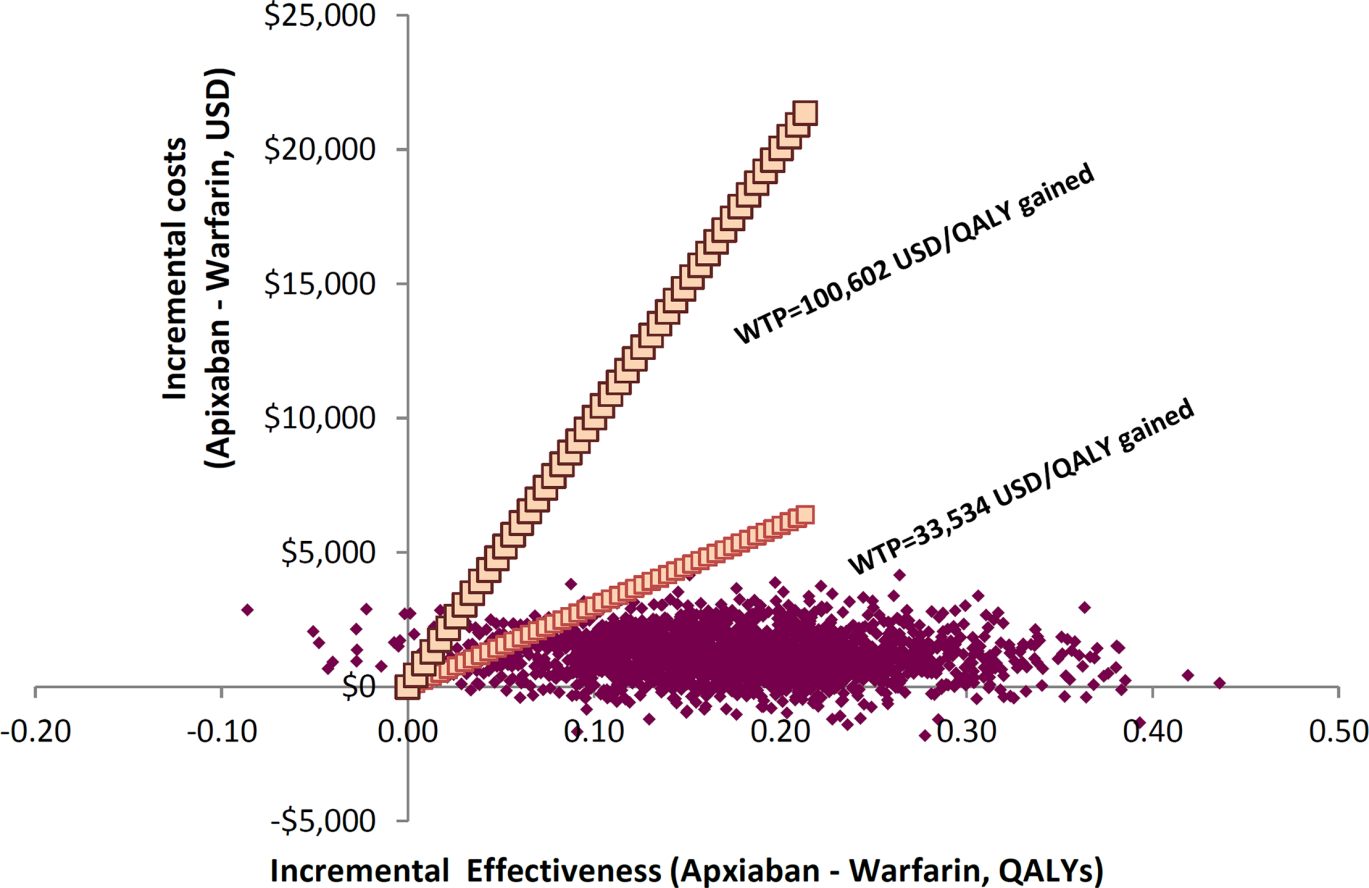
Probabilistic sensitivity analysis of apxiaban versus warfarin.

The probabilistic sensitivity analysis demonstrated that apixiban is more cost-effective against warfarin over a life-time horizon (**Fig 3**). ICER was below the threshold of USD 33,534 per QALY (one local GDP per capita) in 96% of the trials. When the upper range of the WHO threshold of USD 100,602 per QALY gained (three-times of local GDP per capita) was applied, 98% of the trials supported the cost-effectiveness of apixaban.

**Fig 4:**
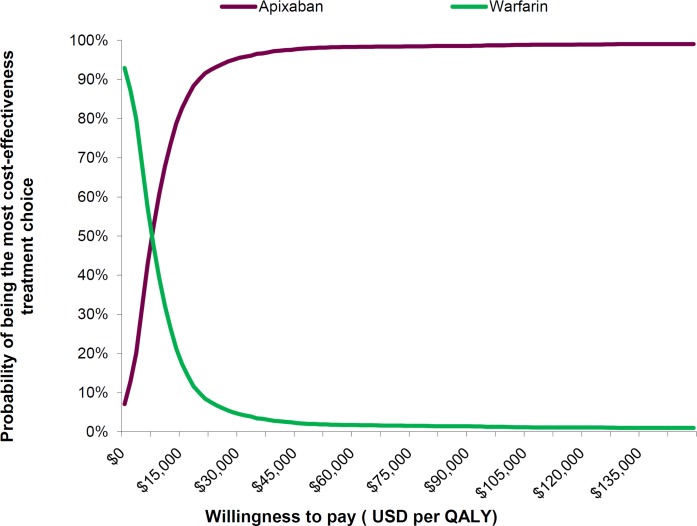
Cost-effectiveness acceptability curves of apixaban and warfarin.

The uncertainties in the cost-effective estimates are further summarised in the cost-effectiveness acceptability curve in **Fig 4.** This demonstrates that apixaban was a superior treatment choice representing the maximum net benefit over warfarin, when payers are willing to pay USD 7,500 or above for one QALY gained.

## Discussion

### Summary of findings

In this study, our principal findings were as follows: (i) fewer thromboembolic events were estimated for the apixaban group in the lifetime cohort simulations, resulting in corresponding increased LYs, QALYs and reduced event-related healthcare costs; (ii) compared with warfarin, apixaban presented an ICER of USD 1,061–14,867 per QALY gained which is within the common acceptable WTP of one local GDP per capita as recommended by the WHO[28]; (iii) both deterministic and probabilistic sensitivity analyses support the cost-effectiveness of apixaban over a range of uncertainties. This study utilises the strength of CDARS, which collects clinical data in the public healthcare setting across the whole territory. As far as we are aware, this is the only study which utilises real-life, Hong Kong-specific cost, patient demographics and clinical profiles, treatment patterns and case fatality rates to compare the cost-effectiveness of apixaban versus warfarin.

The greater health benefits and cost-effectiveness of apixaban findings in this study are in line with other modeling CE studies conducted in western developed countries [8–12]. Lee et al demonstrated apxiaban as a dominant strategy (both cost and life year savings) from the US Medicare perspective [12], while the remaining four studies all found apixaban more cost-effective against warfarin in Australia, Netherlands, Sweden and United Kingdom at ICERs of AUD 13,679[11], EUR 10,576[8], SEK 33,458[9], GBP 11,909[10] per QALY respectively, applying locally relevant WTP thresholds.

### Asian data

In light of the widely reported cost-effectiveness of apixaban in Caucasians, evaluation of the Asian population is still limited. However, from a clinical perspective, Asian and Western patients with AF are not necessarily the same. Firstly, among the five components of the CHADS_2_ (congestive heart failure, hypertension, age≥75 years, diabetes mellitus and stroke) score, similar risk factor profiles were shared, except previous stroke is more prevalent in Asians (29% vs. 18%) [13]. Secondly, stroke risk is higher in Asians when treated with warfarin as reflected in the subgroup analysis of ARISTOTLE trial (3.4% vs. 1.4%) [6, 7]. Thirdly, despite a lower INR in general, warfarin users in Asia are more likely to bleed than non-Asians (3.8% vs. 3.0%) [6, 7]. Although the mechanism for these differences remains to be determined, they may all challenge the proper use and cost-effectiveness of warfarin and its alternatives in Asia. For the first time, our analysis showed that apixaban is cost-effective in a Chinese population. This is an important ethnic group in Asia with a high prevalence of stroke [29] and rapidly increasing ageing population [30], which highlights the prominence of this study.

### Real-life data

Another important differentiator and strength of our study is the use of real-life, local INR control data in our model. It is well documented in the published literature that good INR control, although important for good clinical outcome [31], is unusual in both clinical trial [32] and real-life settings [33]. The ARISTOTLE trial reported that only 50% of warfarin patients have TTR ≥ 66.0% [6]. Hong Kong practice is not an exception. Consistent with our findings from the CDARS retrospective cohort study, which found a median TTR of 40.9%, Ho et al also reported unsatisfactory INR control among patients in Hong Kong with similar TTR (median: 38.8%) [33]. As TTR is negatively correlated with bleeding and thromboembolic events [34], suboptimal INR control in real-life may worsen the performance of warfarin [35]. Potential health benefits offered by apixaban may be greater than that in the clinical trial setting for patients with poor TTR [13] when compared to warfarin. By incorporating real-life INR control for event risks adjustment, our study provides a more realistic picture compared to previous studies where the risks were based on clinical trials [11, 12] or tested in scenario analyses [9, 10].

Of note, variations in healthcare system and services have been well recognised worldwide [36] and this is another key reason why country-specific CEA needs to be conducted. This study summarised local relevant information and will contribute to anticoagulation policy consideration in Hong Kong and other Asian countries with similar healthcare systems.

### Limitations

There are limitations in this modeling analysis. Firstly, clinical efficacy and safety parameters were based on the landmark trial with 1.8 years of follow-up. They were assumed to be constant over the lifetime in the model, but might not reflect the long-term performance of treatments in real terms. Secondly, following a major bleeding event, treatment options were limited to switching to aspirin or restarting the original anticoagulant in 6 weeks. In real-life clinical practice, some patients may cease treatment or choose other anticoagulants. However, aspirin is perceived as a safer alternative to oral anticoagulants in the Chinese population [20] given the particular concerns of increased bleeding risk among the Chinese [37–40]. As indicated in our previous work [41], more than twice the number of NVAF patients was receiving antiplatelet drugs including aspirin, compared to oral anticoagulants in Hong Kong, despite having a higher risk of stroke (CHADS_2_≥2). In addition, the cut-off point of assessing treatment switch was restrict to 90 days, based on Witt et al’s study on the association of warfarin interruption and gastrointestinal tract bleeding [42]. This may be arbitrary as there are no published guidelines on the optimal anticoagulation approach for those experiencing a thromboembolic event at the time our study was conducted [43]. Thirdly, in the baseline analysis, we optimistically assumed no renal monitoring was required for patients on apixaban based on the subgroup analysis of ARISTOTLE [44] which may not be the case in real clinical practice. The subgroup analysis was pre-specified for patients with impaired renal function and showed that apixaban was more effective than warfarin in preventing stroke or systemic embolism and reducing mortality, irrespective of renal function. Considering the median age of 78.9 years in this cohort of patients, we analysed two other scenarios—assuming 50% and 100% of patients will have their renal function evaluated annually, respectively, whilst on apixaban. Both scenarios show that the frequency of renal monitoring had very limited impact on the ICERs (USD 7,270 and USD 7,482 per QALY saved respectively) therefore baseline conclusions remains robust. Fourthly, treatment attributes such as convenience of drug administration, food and drug interaction were not included in this model, which may underestimate the benefits of apixaban. Lastly, the estimate for acute event cost per episode was restricted to general ward charges only and the costs of emergency and intensive care or rehabilitation have not been included. These would have led to underestimates of event related costs for both groups.

## Conclusion

In conclusion, the current comprehensive modeling assessment based on real-life, local clinical, economic evidence and ARISTOTLE trial, demonstrate that apixaban is a cost-effective alternative against warfarin for the prevention of stroke and bleeding events in Chinese patients with NVAF. Our results suggest that apixaban is relatively more favourable in terms of health benefits and incremental cost.

## Supporting Information

S1 File **Figure A. Schematic representation of Markov model.** NVAF: non-valvular atrial fibrillation. **Figure B. Health states transitions from NVAF.** CRNM Bleed: Clinically relevant non-major bleed; ICH: Intracerebral haemorrhage; NVAF: Non-valvular atrial fibrillation; NVAF without original anticoagulation: patients with NVAF who discontinue the initially assigned anticoagulation treatment but have not yet experienced any of the events including ischaemic stroke, haemorrhagic stroke, myocardial infarction, systematic embolism or death. **Table A. Base-case and sensitivity analyses inputs for clinical events, deaths, post-event treatment and anticoagulation management. Table B. Base-case and sensitivity analyses inputs for utility and cost. Table C. The ICD-9 codes (diagnosis codes unless other specified) used in this study. Supplemental References.**
(DOCX)
