# Supplementary material for: Cost-Effectiveness of Apixaban versus Warfarin in Chinese Patients with Non-Valvular Atrial Fibrillation: A Real-Life and Modelling Analyses
Source: PLoS One. 2016 Jun 30;11(6):e0157129. doi: 10.1371/journal.pone.0157129 (PMC4928891; doi:10.1371/journal.pone.0157129)
Supplement: S1 File — Figure A. Schematic representation of Markov model. NVAF: non-valvular atrial fibrillation. Figure B. Health states transitions from NVAF. CRNM Bleed: Clinically relevant non-major bleed; ICH: Intracerebral haemorrhage; NVAF: Non-valvular atrial fibrillation; NVAF without original anticoagulation: patients with NVAF who discontinue the initially assigned anticoagulation treatment but have not yet experienced any of the events including ischaemic stroke, haemorrhagic stroke, myocardial infarction, systematic embolism or death. Table A. Base-case and sensitivity analyses inputs for clinical events, deaths, post-event treatment and anticoagulation management. Table B. Base-case and sensitivity analyses inputs for utility and cost. Table C. The ICD-9 codes (diagnosis codes unless other specified) used in this study. Supplemental References. (DOCX) [file pone.0157129.s001.docx]

**Supporting information**

**Figure A. Schematic representation of Markov model.**


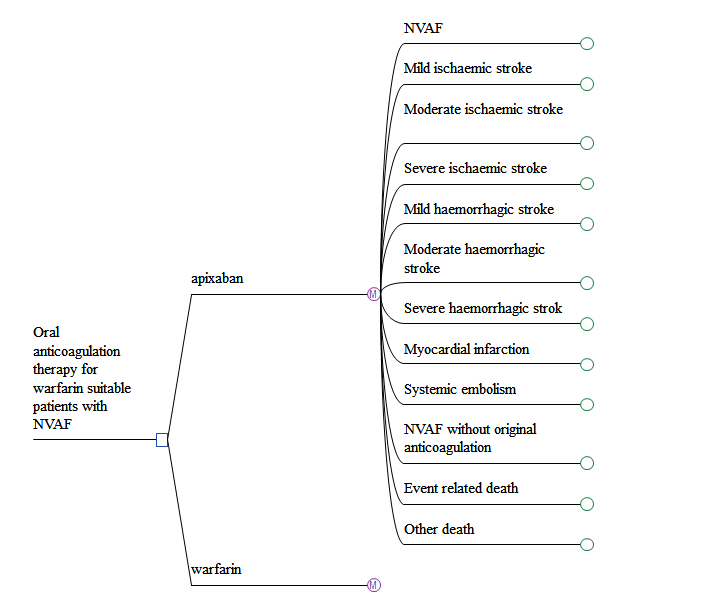


**Figure B. Health states transitions from NVAF.**


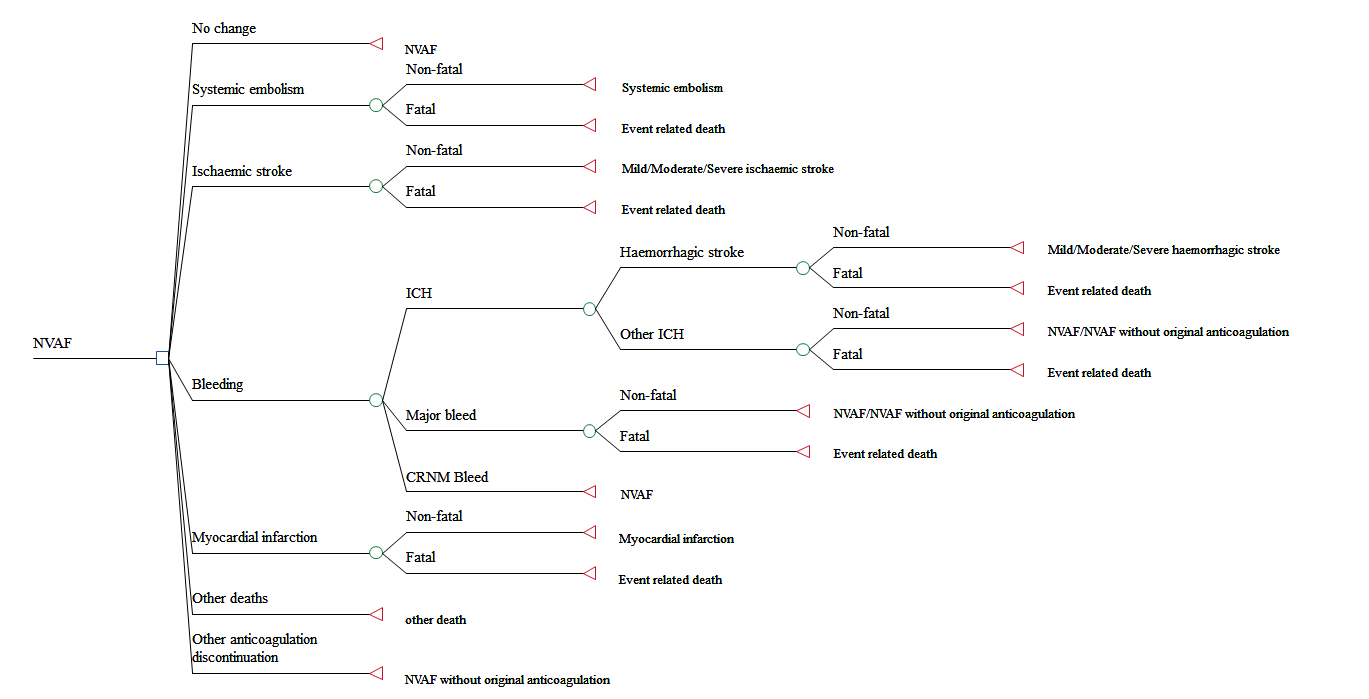


**Table A. Base-case and sensitivity analyses inputs for clinical events, deaths, post-event treatment and anticoagulation management.**

| Variables | Apixaban | | | Warfarin | | |
| --- | --- | --- | --- | --- | --- | --- |
|  | Base-case | Range | References | Base-case | Range | References |
| ***Risk of clinical events*** |  |  |  |  |  |  |
| *Ischaemic Stroke (IS)* |  |  |  |  |  |  |
| Risk of IS (Rate/100 PYs) | 0.98 | 0.56-1.51 | Secondary analysis of ARISTOTLE data | 1.09 | 0.86-1.32 | [1] |
| Risk adjustment factor for IS per decade | 1.40 | 0.80-2.16 | [2] | 1.40 | 0.80-2.16 | [2] |
| Risk of recurrent IS | 4.10 | 3.41-4.91 | [3] | 4.10 | 3.41-4.91 | [3] |
| *Intracranial Haemorrhage(ICH)* |  |  |  |  |  |  |
| Risk of ICH (Rate/100 PYs) | 0.33 | 0.15-0.51 | [1] | 0.80 | 0.35-1.87 | [1] |
| Risk adjustment factor for ICH per decade | 1.97 | 1.79-2.16 | [4] | 1.97 | 1.79-2.16 | [4] |
| Risk of recurrent haemorrhagic stroke | 3.00 | 2.02-4.46 | [3] | 3.00 | 2.02-4.46 | [3] |
| Proportion of haemorrhagic strokes (HS) among ICHs | 0.77 | 0.65-0.87 | Secondary analysis of ARISTOTLE data | 0.64 | 0.44-0.70 | Secondary analysis of ARISTOTLE data |
| *Other major bleeds* |  |  |  |  |  |  |
| Risk of other major bleeds (Rate/100 PYs) | 1.79 | 1.02-2.77 | [1] | 2.27 | 1.30-3.51 | [1] |
| Risk adjustment factor for other major bleeds per decade | 1.97 | 1.79-2.16 | [4] | 1.97 | 1.79-2.16 | [4] |
| Proportion of GI bleeds among other major bleeds | 0.38 | 0.32-0.44 | Assumption (same distribution as observed in ARISTOTLE [1]) | 0.50 | - | CDARS |
| *Clinically relevant non major bleeds (CRNB)* |  |  |  |  |  |  |
| Risk of CRNB (Rate/100 PYs) | 2.08 | 1.19-3.22 | Secondary analysis of ARISTOTLE data | 3.00 | 2.59-3.45 | Secondary analysis of ARISTOTLE data |
| Risk adjustment factor for CRNB per decade | 1.97 | 1.79-2.16 | [4] | 1.97 | 1.79-2.16 | [4] |
| *Myocardial infarction (MI)* |  |  |  |  |  |  |
| Risk of MI for (Rate/100 PYs) | 0.53 | 0.30-0.82 | [1] | 0.61 | 0.45-0.80 | [1] |
| Risk adjustment factor for MI per decade | 1.30 | - | [5] | 1.30 | - | [5] |
| *Other cardiovascular (CV) hospitalisation* |  |  |  |  |  |  |
| Risk of other CV hospitalisation for apixaban (Rate/100 PYs) | 10.46 | 5.98-16.71 | Assumption^*^ | 10.46 | 5.98-16.71 | Assumption^*^ |
| *Systematic Embolism (SE)* |  |  |  |  |  |  |
| Risk of SE (Rate/100 PYs) | 0.09 | 0.05-0.14 | [1] | 0.10 | 0.05-0.20 | Assumption |
| ***Death*** |  |  |  |  |  |  |
| *Case fatality rate (CFR, %)* |  |  |  |  |  |  |
| CFR of IS | 18% | 11-26% | Secondary analysis of AVERROES and ARISTOTLE | 15% | 9-22% | Secondary analysis of AVERROES and ARISTOTLE |
| CFR of HS | 35% | 20-52% | Secondary analysis of AVERROES and ARISTOTLE | 53% | 41-65% | Secondary analysis of AVERROES and ARISTOTLE |
| CFR of other ICH | 13% | 6-22% | Secondary analysis of AVERROES and ARISTOTLE | 13% | 6-22% | Secondary analysis of AVERROES and ARISTOTLE |
| CFR of GI bleeds | 1% | 0-2% | Assumption (same rate as that of warfarin estimated from CDARS) | 1% | 0-2% | CDARS |
| CFR of SE | 9% | 2-21% | ARISTOTLE Case Study Report | 9% | 2-21% | ARISTOTLE Case Study Report |
| CFR of MI for females | 16% | 9-24% | Assumption (same rate as that of warfarin estimated from CDARS) | 16% | 9-24% | CDARS |
| CFR of MI for males | 14% | 8-21% | Assumption (same rate as that of warfarin estimated from CDARS) | 14% | 8-21% | CDARS |
| *Mortality* |  |  |  |  |  |  |
| Background mortality risk | 1 | - | Assumption | 1 | - | Assumption |
| Additional mortality risk adjustment for AF | 1.34 | 1.20-1.53 | [6] | 1.34 | 1.20-1.53 | [6] |
| Additional mortality risk adjustment for mild ischaemic stroke | 3.18 | 1.42-4.94 | [7-9] | 3.18 | 1.42-4.94 | [7-9] |
| Additional mortality risk adjustment for moderate ischaemic stroke | 5.84 | 4.08-7.60 | [7-9] | 5.84 | 4.08-7.60 | [7-9] |
| Additional mortality risk adjustment for severe ischaemic stroke | 15.75 | 13.99-17.51 | [7-9] | 15.75 | 13.99-17.51 | [7-9] |
| Additional mortality risk adjustment for mild haemorrhagic stroke | 3.18 | 1.82-4.92 | [7-9] | 3.18 | 1.82-4.92 | [7-9] |
| Additional mortality risk adjustment for moderate haemorrhagic stroke | 5.84 | 3.34-9.03 | [7-9] | 5.84 | 3.34-9.03 | [7-9] |
| Additional mortality risk adjustment for severe haemorrhagic stroke | 15.75 | 9.00-24.35 | [7-9] | 15.75 | 9.00-24.35 | [7-9] |
| Additional mortality risk adjustment for SE | 1.34 | 1.20-3.18 | Assumption | 1.34 | 1.20-3.18 | Assumption |
| Additional mortality risk adjustment for MI females | 4.16 | 3.44-5.03 | [10] | 4.16 | 3.44-5.03 | [10] |
| Additional mortality risk adjustment for MI males | 2.56 | 2.27-2.88 | [10] | 2.56 | 2.27-2.88 | [10] |
| *Death rate in different treatment arm (same as trial period)* |  |  |  |  |  |  |
| Rate of death (Rate/100 PYs) | 3.08 | 2.50-3.72 | Secondary analysis of ARISTOTLE data | 3.34 | 2.71-4.04 | Secondary analysis of ARISTOTLE data |
| ***Anticoagulation treatment choice post events*** |  |  |  |  |  |  |
| Post ICH (% of switch treatment, aspirin as 2^nd^ line treatment) | 44% | 31-57% | [11] | 44% | 31-57% | [11] |
| Post GI bleeding (% of switch treatment, aspirin as 2^nd^ line treatment | 25% | 1-69% | [12] | 29% | 3-69% | CDARS |
| Post non-ICH and non-GI bleeding (% of switch treatment, aspirin as 2^nd^ line treatment | 25% | - | [12] | 20% | - | CDARS |
| Post IS (% of no changes) | 100% | - | Expert opinion | 100% | - | Assumption |
| Post SE (% of no changes) | 100% | - | Expert opinion | 100% | - | Expert opinion |
| ***Risk for aspirin as 2nd line treatment (Rate/100 PYs)*** |  |  |  |  |  |  |
| Risk for IS | 3.45 | 1.97-5.34 | Secondary data analysis of AVERROES | 3.45 | 1.97-5.34 | Secondary data analysis of AVERROES |
| Risk of ICH | 0.32 | 0.18-0.50 | Secondary data analysis of AVERROES | 0.32 | 0.18-0.50 | Secondary data analysis of AVERROES |
| Risk of other major bleeds | 0.89 | 0.51-1.37 | Secondary data analysis of AVERROES | 0.89 | 0.51-1.37 | Secondary data analysis of AVERROES |
| Risk of CRNMB | 2.94 | 1.68-4.54 | Secondary data analysis of AVERROES | 2.94 | 1.68-4.54 | Secondary data analysis of AVERROES |
| Risk of MI | 1.11 | 0.63-1.72 | Secondary data analysis of AVERROES | 1.11 | 0.63-1.72 | Secondary data analysis of AVERROES |
| Risk of other CV hospitalisation | 13.57 | 7.76-20.98 | Secondary data analysis of AVERROES | 13.57 | 7.76-20.98 | Secondary data analysis of AVERROES |
| ***Anticoagulation management*** |  |  |  |  |  |  |
| *Patients experiencing dyspepsia whilst on treatment (%)* | 1.67% | - | ARISTOTLE Case Study Report | 0.41% | - | CDARS |
| *Patients requiring annual renal monitoring whilst on treatment (%)* | 0 | 0-100% | Assumption based on Secondary analysis of ARISTOTLE data | 29.65% | - | CDARS |

Footnote: * rate for apixaban taken from the AVERROES, assume same rate for warfarin;

Abbreviations: ARISTOTLE: Apixaban for Reduction in Stroke and Other Thromboembolic Events in Atrial Fibrillation; AVERROES：Apixaban Versus Acetylsalicylic Acid (ASA) to Prevent Stroke in Atrial Fibrillation Patients Who Have Failed or Are Unsuitable for Vitamin K Antagonist Treatment; CDARS：Clinical Data Analysis and Reporting System of Hong Kong

**Table B. Base-case and sensitivity analyses inputs for utility and cost**

| Variables | Basecase | Ranges | References |
| --- | --- | --- | --- |
| ***Utility*** |  |  |  |
| Utility of AF | 0.76 | 0.74-0.77 | [13] |
| Utility of mild ischaemic stroke | 0.62 | 0.56-0.67 | [14] |
| Utility of moderate ischaemic stroke | 0.56 | 0.51-0.62 | [14] |
| Utility of severe ischaemic stroke | 0.51 | 0.46-0.57 | [14] |
| Utility of mild haemorrhagic stroke | 0.62 | 0.56-0.67 | [14] |
| Utility of moderate haemorrhagic stroke | 0.56 | 0.51-0.62 | [14] |
| Utility of severe haemorrhagic stroke | 0.51 | 0.46-0.57 | [14] |
| Utility of SE | 0.63 | 0.59-0.66 | [14] |
| Utility decrement of MI | 0.61 | 0.57-0.65 | [14] |
| Utility decrement of other ICH | 0.15 | 0.08-0.24 | [14] |
| Utility decrement of other major bleed | 0.15 | 0.08-0.24 | [14] |
| Utility decrement of CRNMB | 0.06 | 0.03-0.10 | [14] |
| Utility decrement of other CV hospitalisation | 0.13 | 0.08-0.18 | [14] |
| Utility decrement while on aspirin (2^nd^ line treatment) | 0 | - | [15] |
| Utility decrement while on warfarin | 0.01 | 0-0.08 | [15] |
| Utility decrement while on apixaban | 0 | - | Assumption |
| Utility decrement while on aspirin (2^nd^ line) | 0 | - | [15] |
| Utility decrement of dyspepsia | 0.2 | - | [16, 17] |
| Utility decrement of renal monitoring | 0 | - | Assumption |
| Utility decrement associated with age | 0.00029 | - | [14] |
| ***Cost*** |  |  |  |
| *Daily drug acquisition cost (USD/day)* |  |  |  |
| Apixaban | 2.21 | 1.77-2.65 | Local market price |
| Aspirin (2^nd^ line) | 0.13 | 0.13-0.16 | Local market price |
| Warfarin | 0.19 | 0.19-0.23 | Local market price |
| *Cost of anticoagulation management, monitoring and routine care* |  |  |  |
| Frequency of INR monitoring of warfarin (number/month) | 1.0 | - | CDARS |
| Cost of INR monitoring (USD/visit) | 19.35 | 16.19-23.12 | [18] |
| Annual cost of routine care visits due to dyspepsia (USD/patient) | 7 | - | CDARS, [18] |
| Cost of routine care visits due to renal monitoring (USD/patient-year) | 19 | - | CDARS, [18] |
| *Acute Event cost (USD/episode)* |  |  |  |
| Mild ischaemic stroke^*^ | 11,715 | 9,372-14,059 | CDARS, [18] |
| Moderate ischaemic stroke^*^ | 11,013 | 8,810-13,215 | CDARS, [18] |
| Severe ischaemic stroke^*^ | 24,251 | 19,401-29,101 | CDARS, [18] |
| Fatal ischaemic stroke^*^ | 15,582 | 12,466-18,698 | CDARS, [18] |
| Mild haemorrhagic stroke^*^ | 10,992 | 8,794-13,190 | CDARS, [18] |
| Moderate haemorrhagic stroke^*^ | 10,332 | 8,266-12,398 | CDARS, [18] |
| Severe haemorrhagic stroke^*^ | 22,753 | 18,202-27,304 | CDARS, [18] |
| Fatal haemorrhagic stroke^*^ | 14,619 | 11,695-17,543 | CDARS, [18] |
| Systemic embolism | 10,809 | 8,647-12,971 | CDARS, [18] |
| Other ICH | 4816 | 3,853-5,779 | [19] |
| GI Bleeds | 7367 | 5,894-8,840 | CDARS, [18] |
| Non-ICH and non-GI bleeding | 4469 | 3,575-5,363 | CDARS, [18] |
| CRNM Bleeds | 4348 | 3,478-5,218 | CDARS, [18] |
| Myocardial infarction | 7790 | 6,232-9,348 | CDARS, [18] |
| Other CV Hospitalisation | 5254 | 4,203-6,305 | CDARS, [18] |
| *Long-term maintenance cost (USD/month, lifetime)* |  |  |  |
| Mild ischaemic stroke | 250 | 200-300 | Assumption^†^ |
| Moderate ischaemic stroke | 271 | 217-325 | Assumption^†^ |
| Severe ischaemic stroke | 766 | 613-919 | Assumption^†^ |
| Mild haemorrhagic stroke | 234 | 187-281 | Assumption^†^ |
| Moderate haemorrhagic stroke | 254 | 203-305 | Assumption^†^ |
| Severe haemorrhagic stroke | 719 | 575-863 | Assumption^†^ |
| Systemic embolism | 230 | 184-276 | Assumption^†^ |
| Myocardial infarction | 25 | 20-30 | Assumption‡ |

Footnote: *stroke distribution assumed to be the same as the ARISTOTLE trial (secondary analysis); ^†^ long-term maintenance cost considered resource use in hospital, primary care, healthcare contacts and utilisation of social services; ‡ long-term maintenance cost considered medication cost including ACE inhibitors, beta blockers and statins.

Abbreviations: ARISTOTLE: Apixaban for Reduction in Stroke and Other Thromboembolic Events in Atrial Fibrillation; CDARS: Clinical Data Analysis and Reporting System of Hong Kong

**Table C. The ICD-9 codes (diagnosis codes unless other specified) used in this study.**

| **ICD-9 codes** | **Descriptions** | |
| --- | --- | --- |
| **Atrial fibrillation** | | |
| 427.3 | Atrial fibrillation and flutter | |
|  |  | |
| **Valvular heart diseases/replacement or hyperthyroidism** | | |
| 242 | Thyrotoxicosis with or without goitre | |
| 394.0 | Mitral stenosis | |
|  |  | |
| Procedures |  | |
| 35.20 | Open And Other Replacement Of Unspecified Heart Valve | |
| 35.22 | Open And Other Replacement Of Aortic Valve | |
| 35.24 | Open And Other Replacement Of Mitral Valve | |
| 35.26 | Open And Other Replacement Of Pulmonary Valve | |
| 35.28 | Open And Other Replacement Of Tricuspid Valve | |
|  |  | |
| **Congestive Heart Failure** | | |
| 398.91 | Rheumatic heart failure (congestive) | |
| 402.01 | Malignant hypertensive heart disease with heart failure | |
| 402.11 | Benign hypertensive heart disease with heart failure | |
| 402.91 | Unspecified hypertensive heart disease with heart failure | |
| 404.01 | Hypertensive heart and chronic kidney disease, malignant, with heart failure and with chronic kidney disease stage I through stage IV, or unspecified | |
| 404.03 | Hypertensive heart and chronic kidney disease, malignant, with heart failure and with chronic kidney disease stage V or end stage renal disease | |
| 404.11 | Hypertensive heart and chronic kidney disease, benign, with heart failure and with chronic kidney disease stage I through stage IV, or unspecified | |
| 404.13 | Hypertensive heart and chronic kidney disease, benign, with heart failure and chronic kidney disease stage V or end stage renal disease | |
| 404.91 | Hypertensive heart and chronic kidney disease, unspecified, with heart failure and with chronic kidney disease stage I through stage IV, or unspecified | |
| 404.93 | Hypertensive heart and chronic kidney disease, unspecified, with heart failure and chronic kidney disease stage V or end stage renal disease | |
| 428 | Heart failure | |
|  |  | |
| **Hypertension** | | |
| 401 | Essential hypertension | |
| 402 | Hypertensive heart disease | |
| 403 | Hypertensive chronic kidney disease | |
| 404 | Hypertensive heart and chronic kidney disease | |
| 405 | Secondary hypertension | |
| 437.2 | Hypertensive encephalopathy | |
|  |  | |
| **Diabetes** |  | |
| 250 | Diabetes mellitus | |
|  |  | |
| **Ischaemic stroke** | | |
| 433.01 | Occlusion and stenosis of basilar artery with cerebral infarction | |
| 433.11 | Occlusion and stenosis of carotid artery with cerebral infarction | |
| 433.21 | Occlusion and stenosis of vertebral artery with cerebral infarction | |
| 433.31 | Occlusion and stenosis of multiple and bilateral precerebral arteries with cerebral infarction | |
| 433.81 | Occlusion and stenosis of other specified precerebral artery with cerebral infarction | |
| 433.91 | Occlusion and stenosis of unspecified precerebral artery with cerebral infarction | |
| 434 | Occlusion of cerebral arteries | |
| 436 | Acute, but ill-defined, cerebrovascular disease | |
| 437.0 | Cerebral atherosclerosis | |
| 437.1 | Other generalized ischaemic cerebrovascular disease | |
|  |  | |
| **Transient ischaemic attack** | | |
| 435 | Transient cerebral ischaemia | |
| **Systemic embolism** | | |
| 444 | | Arterial embolism and thrombosis |
| 445 | | Atheroembolism |
|  | |  |
| **Myocardial infarction** | | |
| 410 | | Acute myocardial infarction |
|  | |  |
| **Intracranial haemorrhage (haemorrhagic stroke)** | | |
| 430 | | Subarachnoid haemorrhage |
| 431 | | Intracerebral haemorrhage |
| 432 | | Other and unspecified intracranial haemorrhage |
|  | |  |
| **Non-intracranial haemorrhage** | | |
| Gastrointestinal bleeding | | |
| 455.2 | | Internal haemorrhoids with other complication |
| 455.5 | | External haemorrhoids with other complication |
| 455.8 | | Unspecified haemorrhoids with other complication |
| 456.0 | | Oesophageal varices with bleeding |
| 456.2 | | Oesophageal varices in diseases classified elsewhere |
| 530.7 | | Mallory—Weiss syndrome |
| 530.8 | | Oesophageal disorder nec |
| 531.0 | | Acute gastric ulcer with haemorrhage |
| 531.2 | | Acute gastric ulcer with haemorrhage and perforation, without mention of obstruction |
| 531.4 | | Chronic or unspecified gastric ulcer with haemorrhage |
| 531.6 | | Chronic or unspecified gastric ulcer with haemorrhage and perforation |
| 532.0 | | Acute duodenal ulcer with haemorrhage |
| 532.2 | | Acute duodenal ulcer with haemorrhage and perforation |
| 532.4 | | Chronic or unspecified duodenal ulcer with haemorrhage |
| 532.6 | | Chronic or unspecified duodenal ulcer with haemorrhage and perforation |
| 533.0 | | Acute peptic ulcer of unspecified site with haemorrhage |
| 533.2 | | Acute peptic ulcer of unspecified site with haemorrhage and perforation |
| 533.4 | | Chronic or unspecified peptic ulcer of unspecified site with haemorrhage |
| 533.6 | | Chronic or unspecified peptic ulcer of unspecified site with haemorrhage and perforation |
| 534.0 | | Acute gastrojejunal ulcer with haemorrhage |
| 534.2 | | Acute gastrojejunal ulcer with haemorrhage and perforation, without mention of obstruction |
| 534.4 | | Chronic or unspecified gastrojejunal ulcer with haemorrhage |
| 534.6 | | Chronic or unspecified gastrojejunal ulcer with haemorrhage and perforation |
| 535.01 | | Acute gastritis, with haemorrhage |
| 535.11 | | Atrophic gastritis, with haemorrhage |
| 535.21 | | Gastric mucosal hypertrophy, with haemorrhage |
| 535.31 | | Alcoholic gastritis, with haemorrhage |
| 535.41 | | Other specified gastritis, with haemorrhage |
| 535.51 | | Unspecified gastritis and gastroduodenitis, with haemorrhage |
| 535.61 | | Duodenitis, with haemorrhage |
| 535.71 | | Eosinophilic gastritis, with haemorrhage |
| 537.8 | | Gastroduodenal dis nec |
| 562.02 | | Diverticula sm intestine w haemorrhage |
| 562.03 | | Diverticulitis sm intestine w haemorrhage |
| 562.12 | | Diverticula of colon w haemorrhage |
| 562.13 | | Diverticulitis of colon w haemorrhage |
| 568.81 | | Hemoperitoneum |
| 569.3 | | Rectal and anal haemorrhage |
| 569.85 | | Angiodysplasia with hem nec |
| 578 | | Gastrointest haemorr |
|  | |  |
| Other sites | |  |
| 423.0 | | Hemopericardium |
| 459.0 | | Haemorrhage NOS |
| 593.81 | | Renal vascular disorder |
| 599.7 | | Haematuria |
| 623.8 | | Noninflam dis vagina nec |
| 626.2 | | Excessive menstruation |
| 626.6 | | Metrorrhagia |
| 719.1 | | Hemarthrosis |
| 784.7 | | Epistaxis |
| 784.8 | | Haemorrhage from throat |
| 786.3 | | Haemoptysis |
|  | |  |
| Dyspepsia | |  |
| 536.8 | | Dyspepsia and other disorders of function of stomach |

**Supplemental references**

[1] Granger CB, Alexander JH, McMurray JJV, et al. Apixaban versus Warfarin in Patients with Atrial Fibrillation. New Engl J Med. 2011;365:981-92.

[2] Risk factors for stroke and efficacy of antithrombotic therapy in atrial fibrillation. Analysis of pooled data from five randomized controlled trials. Archives of internal medicine. 1994;154:1449-57.

[3] Mohan KM, Crichton SL, Grieve AP, Rudd AG, Wolfe CD, Heuschmann PU. Frequency and predictors for the risk of stroke recurrence up to 10 years after stroke: the South London Stroke Register. Journal of neurology, neurosurgery, and psychiatry. 2009;80:1012-8.

[4] Ariesen MJ, Claus SP, Rinkel GJ, Algra A. Risk factors for intracerebral hemorrhage in the general population: a systematic review. Stroke. 2003;34:2060-5.

[5] Executive Summary of The Third Report of The National Cholesterol Education Program (NCEP) Expert Panel on Detection, Evaluation, And Treatment of High Blood Cholesterol In Adults (Adult Treatment Panel III). Jama. 2001;285:2486-97.

[6] Friberg L, Hammar N, Pettersson H, Rosenqvist M. Increased mortality in paroxysmal atrial fibrillation: report from the Stockholm Cohort-Study of Atrial Fibrillation (SCAF). Eur Heart J. 2007;28:2346-53.

[7] Huybrechts KF, Caro JJ, Xenakis JJ, Vemmos KN. The prognostic value of the modified Rankin Scale score for long-term survival after first-ever stroke. Results from the Athens Stroke Registry. Cerebrovasc Dis. 2008;26:381-7.

[8] Henriksson KM, Farahmand B, Johansson S, Asberg S, Terent A, Edvardsson N. Survival after stroke--the impact of CHADS2 score and atrial fibrillation. Int J Cardiol. 2010;141:18-23.

[9] Bronnum-Hansen H, Davidsen M, Thorvaldsen P. Long-term survival and causes of death after stroke. Stroke. 2001;32:2131-6.

[10] Bronnum-Hansen H, Jorgensen T, Davidsen M, et al. Survival and cause of death after myocardial infarction: the Danish MONICA study. Journal of clinical epidemiology. 2001;54:1244-50.

[11] Claassen DO, Kazemi N, Zubkov AY, Wijdicks EF, Rabinstein AA. Restarting anticoagulation therapy after warfarin-associated intracerebral hemorrhage. Arch Neurol. 2008;65:1313-8.

[12] Sorensen SV, Dewilde S, Singer DE, Goldhaber SZ, Monz BU, Plumb JM. Cost-effectiveness of warfarin: trial versus "real-world" stroke prevention in atrial fibrillation. American heart journal. 2009;157:1064-73.

[13] Ho JCS, Chang AM, Yan BP, Yu CM, Lam YY, Lee VWY. Dabigatran Compared With Warfarin for Stroke Prevention With Atrial Fibrillation: Experience in Hong Kong. Clinical cardiology. 2012;35:E40-E5.

[14] Sullivan PW, Slejko JF, Sculpher MJ, Ghushchyan V. Catalogue of EQ-5D scores for the United Kingdom. Medical decision making : an international journal of the Society for Medical Decision Making. 2011;31:800-4.

[15] Gage BF, Cardinalli AB, Owens DK. The effect of stroke and stroke prophylaxis with aspirin or warfarin on quality of life. Archives of internal medicine. 1996;156:1829-36.

[16] Mahadeva S, Wee HL, Goh KL, Thumboo J. The EQ-5D (Euroqol) is a valid generic instrument for measuring quality of life in patients with dyspepsia. BMC gastroenterology. 2009;9:20.

[17] Mahadeva S, Yadav H, Rampal S, Goh K-L. Risk Factors Associated With Dyspepsia in a Rural Asian Population and Its Impact on Quality of Life. Am J Gastroenterol. 2010;105:904-12.

[18] Hospital Authority of Hong Kong. Hospital Authority Ordinance (Chapter 113): Revisons to list of charges. Available at: <http://www.ha.org.hk/visitor/ha_visitor_index.asp?Content_ID=10045&Lang=ENG>. Accessed October 15^th^, 2014.

[19] Department of Health TUK. 2009-10 reference costs publication 2009-10.
